# Changes in food quality and characterization under thermal accumulation conditions during Chinese cooking

**DOI:** 10.1002/fsn3.3908

**Published:** 2023-12-29

**Authors:** Mingzan Zhang, Yun Yang, Hongwen Zhang, Cuiqin Li, Laping He, Li Deng

**Affiliations:** ^1^ Guizhou Industry Polytechnic College Guiyang P.R. China; ^2^ Key Laboratory of Agricultural and Animal Products Store & Processing of Guizhou Province Guiyang P.R. China; ^3^ College of Liquor and Food Engineering Guizhou University Guiyang P.R. China; ^4^ School of Chemistry and Chemical Engineering Guizhou University Guiyang P.R. China

**Keywords:** Chinese cooking, superheat value, thermal accumulation, unsteady heat transfer

## Abstract

Chinese cooking is the primary treatment method for table food in China. The process is complex and large‐scale, which is important to the macroeconomy and national nutrition and health. First, this article puts forward the concept of thermal accumulation for Chinese cooking by taking pork tenderloin fried at different oil temperatures, explaining changes in moisture content, hardness, and color with different thermal accumulation conditions, and measuring kinetic parameters. The variations of L* and b* obtained by the experimental results belong to the first‐order reaction kinetic model, while the changes in water content and shear force belong to the zero‐order reaction kinetic model. Simultaneously, the superheat value is used as a thermal accumulation indicator, combined with sensory evaluation to determine that the *Z* value of the human sensory overheating of pork tenderloin is 99°C, and *O*_*s*,max_ (*Z* = 99°C, the reference temperature is 110°C) is 5.86 min.

## Graphical abstract

The key finding of the work: The definition of heat accumulation was first proposed in Chinese cooking. Thermal accumulation scorching phenomenon mechanism was revealed. Heat accumulation concept is the development of the existing maturity value theory.

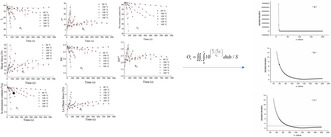

## 1 INTRODUCTION

From ancient history to modern complex forms, Chinese cooking has established its global reputation through traditional Chinese food processing technology. The ultimate goal of cooking is to heat food to maturity to achieve the best eating quality (Huang, 2016). Our previous work (Deng, 2013a) established a representative control equation for heat/mass transfer accounting for mass and energy balance during cooking, and proposed a value theory. Notably, the heat transfer from the surface of the food particles to the center during the cooking process is a non‐steady‐state process. Unsteady heat transfer is the primary technical feature of Chinese cooking. The temperature distribution at each position of the food particles is significantly different due to the different thermal resistance, resulting in different cooking characteristics.

Due to the non‐steady‐state heat transfer during the cooking process, the cooking quality is not uniform, and the surface may deteriorate due to overheating (Ahrné et al., 2007) like browning the meat. In this context, Our previous work (Deng, 2013b) proposed a superheat value (*O* value) to characterize the equivalent heating time of the overheated cooking quality factor relative to the reference temperature. Thus, when the average mature value is reached by the center or volume of the food, it will eventually overheat on the surface and its vicinity, resulting in a specific loss of quality. The superheat value is a quantitative expression of the deterioration of cooking quality caused by overheating.

Previous studies have focused on process characteristics such as center maturity. Heat transfer analysis of the surface of food particles is less involved. We put forward the concept of thermal accumulation because of the hardening phenomenon of food particle surfaces during cooking. During the cooking process, the surface temperature of the unsteady heat transfer food particles reaches the boiling point of the water, and the center does not arrive, forming surface an evaporation interface (Ghaitaranpour et al., 2018). As the cooking process continues, the food surface continues to lose water, and the evaporation interface moves toward the center, leading to an increase in temperature outside the food evaporation interface, which approaches a change in the physical properties of the medium temperature (mainly manifested in hardness) (Purlis & Salvadori, 2010). Simultaneously, caramelization and Maillard reactions occur with a golden yellow color, but both reactions develop non‐enzymatic or non‐oxidative browning with low moisture content, and high temperature can promote it (Ghaitaranpour et al., 2018). The movement of the evaporative interface produced by this process divides the food particles into two regions: the shell and the core. These phenomena occurring in the shell are collectively referred to as thermal accumulation. In short, the heat accumulation phenomenon is primarily a collection of physical changes in the shell color and structure, and the cooking process involves chemical transformations, such as Maillard and caramelization reactions (Kondjoyan et al., 2016). The nature of heat accumulation is due to unsteady heat transfer caused by inconsistent heat transfer rates. Chinese cooking is a type of non‐steady‐state heat transfer, considering that solid food's heat accumulation phenomenon is inevitable.

Food surface cooking quality is crucial for consumers (Pathare & Roskilly, 2016). If the heating is fast in the stir‐frying process, the evaporation interface cannot be formed while the cooking is completed, and the shell becomes fragile (Deng, 2013a). Hence, studying oil stir‐frying and using fried meat to analyze the heat accumulation rule is inappropriate. During deep stir‐frying, the quality kinetics of heat accumulation phenomena (such as brightness L*, yellowness value b*, shear force, water content, etc.) should be considered. At the same time, the superheat value can be used as a heat accumulation index, combined with a sensory evaluation to examine the quality characteristics of Chinese cooking.

This paper analyzed the cooking quality factors under the heat accumulation such as brightness L*, yellowness value b*, shear force, and water content dynamically by taking pork tenderloin fried at different oil temperatures. The kinetic mechanism of the process was discussed, combined with a sensory evaluation test. The present study will explore the essence of heat accumulation by providing a theoretical reference for actual cooking.

## 2 MATERIALS AND METHODS

### 2.1 Materials

Pork Tenderloin was obtained from Heli Supermarket, Huaxi District, Guiyang City, China. Edible palm oil was commercially available.

### 2.2 Instruments and equipment

Slicer (BL658) was from Bole Electronic Appliance Co. Ltd, Shenzhen, China. Colorimeter (HP‐2132) was from Hanpu Optoelectronics Technology Co. Ltd, Shanghai, China; Moisture Analyzer (MB35) from Ohaus International Trading Co. Ltd, Shanghai, China; Computer (Type: E5‐572G‐528R) from Acer Computer (Shanghai) Co., Ltd, China; Digital display muscle tenderness tester (C‐LM3B) from the facility of College of Engineering Northeast Agricultural University, China; Culinary heat transfer and kinetic data acquisition and analysis system was built by Guizhou University, China (Zhou et al., 2013); Adjustable speed constant temperature oil bath (DF‐101T‐5) from Lichen Bangxi Instrument Technology Co., Ltd., Shanghai, China. The S‐type pitot tube and electronic micro pressure gauge (ZCF‐D‐01) were from Shanghai Yiou Instrument Equipment Co., Ltd, China. Thermocouple (WRTK‐103) was from Ningbo Aoqi Automation Instrument Equipment Co., Ltd, China.

**FIGURE 1:**
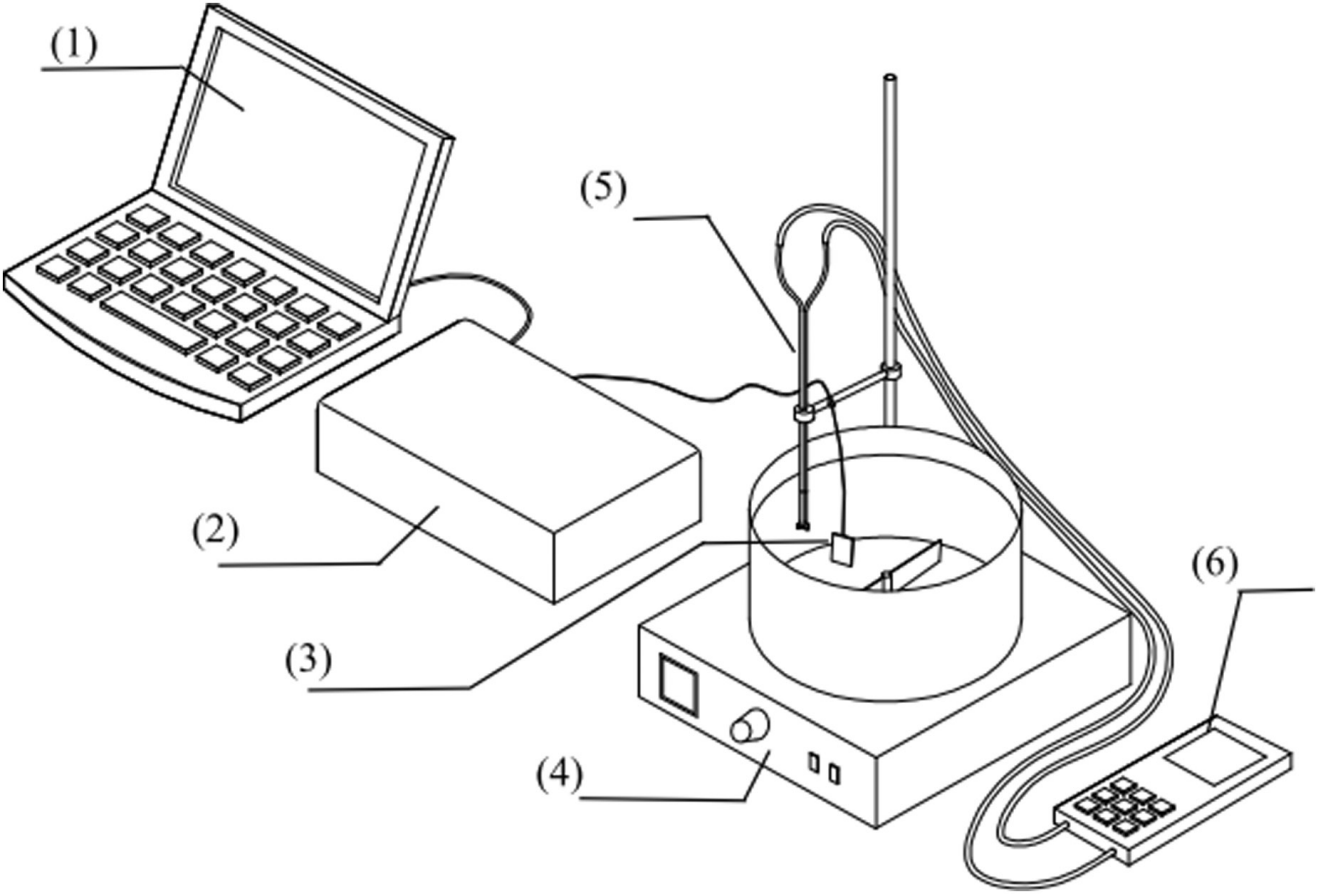
Schematic diagram of the stir‐frying cooking simulation device, (1) Computer, (2) Culinary heat transfer and kinetic data acquisition and analysis system, (3) Thermocouples and test meat slices, (4) Adjustable speed constant temperature oil bath, (5) S‐type Pitot Tube, and (6) Electronic Micro Pressure Gauge.

Stir‐frying uses the stir‐frying cooking simulation device shown in Figure 1, which was carried out in a variable speed and constant temperature oil bath with an oil bath temperature range of 60 to 180°C.

### 2.3 Kinetic test method

#### 2.3.1 Kinetic principle

Kinetics food quality changes during the process transfer reaction determines the cooking quality, which is the core principle of cooking. Changes in cooking quality can be analyzed by food chemistry, food physics, and food microbes. Temperature change and cooking quality are related to heating changes in cooking ingredients (Deng, 2013a). Among them, chemical reaction kinetics is the basic theoretical model. The measured kinetic parameters can be applied to analyze, evaluate, and optimize food processing (Shi et al., 2019). During food processing and storage, most of the food quality changes are based on the zero‐level (*n* = 0) or first‐level (*n* = 1) model (Gao, 2007).

The 0‐order reaction kinetics comes as:

(1) $$A=A_{0}-\mathrm{kt}$$

where *A* is the quality index at time *t*, where the unit is determined by the quality index of the measurement object; *A*_0_ is the initial quality index, and the unit is determined by the quality index of the measurement object; *k* is the reaction rate constant s^−1^.

The 1st order reaction kinetics comes as:

(2) $$\;\ln A=\ln A_{0}-\mathrm{kt}$$

An important basis for speculating the reaction mechanism is the degree of control of reaction order and reaction quality index on the reaction rate. In practical applications, Equations (1) and (2) can be used to obtain the decision coefficient *R* ^2^ of the corresponding reaction and take the reaction series with a larger *R* ^2^.

(3) $$\ln k=-\frac{E_{a}}{\mathrm{RT}}+\ln k_{0}$$

where *R* is the molar gas constant, and *E*_*a*_ is the activation energy of the reaction.

The *D* value is the time required for a logarithmic cycle of food quality to change at a specific temperature in min, and the expression is as follows:

(4) $$D=\frac{2.303}{k*60}$$

The *Z* value can be calculated as the temperature required to change the *D* value of the food quality for a logarithmic cycle (90%), °C, and its expression is:

(5) $$Z=\frac{T_{2}–T_{1}}{\mathrm{lg}D_{1}-\mathrm{lg}D_{2}}$$

The *Z* value reflects the temperature sensitivity of the reaction. Their respective *Z* values determine the rate of increase in the superheat value. The smaller the *Z* value, the more sensitive it is to the temperature and, consequently, a higher increase rate.

The spatial distribution of the temperature is not uniform due to the non‐steady‐state characteristics of the cooking process, and the overheating occurs in different spatial locations. The superheat value (*O* value) for the surface of the particles is combined with the surface area and divided by the total surface area to obtain the expression of the average superheat value of the surface as below:

(6) $$O_{s}=\iint_{s}\int_{0}^{t}10^{\left(\frac{T_{s}-T_{\mathrm{ref}}}{z_{o}}\right)}\text{dtds}/S$$

where *O*_*s*_ is the surface superheat value in min; *Z*_*o*_ is the quality factor of *Z* value that characterizes superheat; $\iint_{s}\mathrm{ds}$ is the surface area integral; *S* is the particle surface area in m^2^; *T*_*s*_ is the surface temperature in °C. *T*_*ref*_ is the reference temperature in °C.

### 2.4 Raw material treatment

The purchased fresh pork tenderloin was kept in the freezer at −18°C. After 8 ~ 10 h, it was cut with a microtome with a dimension of 4 cm × 4 cm × 0.4 cm (length × width × thickness) and placed in a refrigerator at 4°C for about 12 h.

### 2.5 Experimental procedures

A standard thermometer was used to calibrate the temperature of the oil bath and thermocouple. Then, remove the meat slices from the refrigerator and place them at room temperature. When the meat slices are close to room temperature, they will be fried at six different temperatures (80, 100, 120, 140, 160, and 180°C) at different times, and cooled in ice water. The following parameters were measured for the analysis:

#### 2.5.1 Measurement of color difference

The colorimeter was preheated for 30 min and then calibrated with a whiteboard before measuring the parameters. Randomly selected pork loin from different locations that had absorbed surface moisture with filter paper was tested separately. The results were expressed in CIE L* (brightness) and b* (yellow/blue). The L* of the sample represents brightness from black (0) to white (100), while b* represents yellow (+) to blue (−). Each group of samples was measured six times, and the average value was considered for the analysis.

#### 2.5.2 Determination of water content

The moisture analyzer was preheated for half an hour after being turned on and zeroed. Randomly, 4–5 g of uniformly shredded meat pieces were added to the tray, and the measurement was started. Each sample was measured twice, and the average value was taken.

#### 2.5.3 Tenderness determination

The tenderness of the sliced meat was measured using a muscle tenderness meter. The meat slices with intact muscle fibers were cut to a cross‐sectional area of 2 cm × 0.15 cm along the direction of muscle fibers and then placed on the sample loading table to measure the shearing force required to be cut by the probe blade. Each sample was measured three times, and the average value was considered.

### 2.6 Sensory evaluation

To determine the maximum thermal accumulation, as referred to the above results, we re‐selected the test temperatures as 120, 140, 160, and 180°C. Then, 10 samples were selected at the above temperatures and other time intervals, cooled in ice, removed from the water, dried, and professionally trained. Finally, food reviewers had given their feedback (Wang, 2018). Based on the feedback and combined with the quality requirements of fried cooking, a sensory score table was prepared, as shown in Table 1. An unacceptable score is considered to be less than 60. Specific steps are as follows:

1. We prepared the pork tenderloin and took it out according to raw material treatment. When the temperature of the meat slice reached room temperature, a stir‐frying simulation device was used to turn on the maximum flow rate. The heat treatment proportional to time at each set of temperatures is as follows: 120°C (30:30:300 s), 140°C (20:20:200 s), 160°C (15:15:150 s), and 180°C (10:10:100 s), cooled in ice water and dried;
2. Since the flow rate of the oil bath is speedy, the surface temperature can be approximated as the medium temperature. By assuming different *Z* values, Equation (6) can be used to calculate the surface superheat value *O*_*s*_ at each temperature sampling time. At each assumption, with a specific *Z* value, the standard deviation of the surface superheat value converted from the sensory score was calculated. The same process was followed for each temperature with a *Z* value. Finally, the standard deviation corresponding to four temperatures with a *Z* value was averaged as human sense error *σ*(*O*_*s,z*_);
3. The scores were averaged at 120 ~ 180°C, the time t_60_ corresponding to the score 60 was calculated by Matlab fitting interpolation and different *Z* values were assigned to Matlab. Then, we calculated the surface overheat value *O*_*s*_ corresponding to t_60_ at four different temperatures with varying values of *Z*, and then calculated the standard deviation *σ*(*O*_*s*_) of *O*_*s*_ at each *Z* value;
4. First, the *σ*(*O*_*s,z*_) values obtained from (2) averaged and then added this average value with the *Z*–*σ*(*O*_*s*_) values obtained from (3) at the lowest point of intersection. The *Z* value corresponding to the intersection point is the *Z* value of a person's sensory response (heat accumulation) to the pork tenderloin.

**TABLE 1:** Fried cooking sensory evaluation data.

Project	20–17 score	16–13 score	12–9 score	8–5 score	4–1 score
Color and luster	Best color, golden yellow, shiny	Good color, golden yellow, shiny	Fair color, golden yellow	Dark color burnt yellow	Dark color, severely burnt
Mouthfeel	Good chewing, chewy and tender	Easy to chew, good tenderness	Moderate tenderness and delicate meat	The meat is delicate, not easy to chew, and slightly jagged	The meat is rough and not easy to chew, with obvious jaggedness
Smell	With meat aroma, pure odor, and strong aroma	It has a unique aroma of meat	It has the inherent flavor of meat	It has the intrinsic flavor of meat, and the aroma is poor	Slightly poor smell, insufficient aroma, slightly fishy
Taste	Meat‐specific taste, best taste, best aftertaste	It has the unique taste of meat and a moderate taste	Moderate taste, no other odor	General taste, other odors	It has a terrible taste; other odors are obvious
Organizational state	Compact structure and good toughness	Tight organization and best toughness	Tight organization and resilience	Harder organization and poor toughness	The tissue is tough, rough, and non‐tough

### 2.7 Statistical analysis

Data were expressed as mean ± standard deviation. For statistical analysis, Microsoft Excel was used.

## 3 RESULTS AND DISCUSSION

### 3.1 Kinetic analysis of apparent chromaticity change of pork tenderloin shell during the thermal accumulation

Chromaticity parameters L* and b* are critical indicators that reflect the appearance of fried food (Zhang et al., 2016). The kinetic analysis of brightness L* and yellowness b* caused by heat accumulation during stir‐frying is reflected in the experiments.

#### 3.1.1 Kinetic analysis of brightness L* during thermal accumulation

**FIGURE 2:**
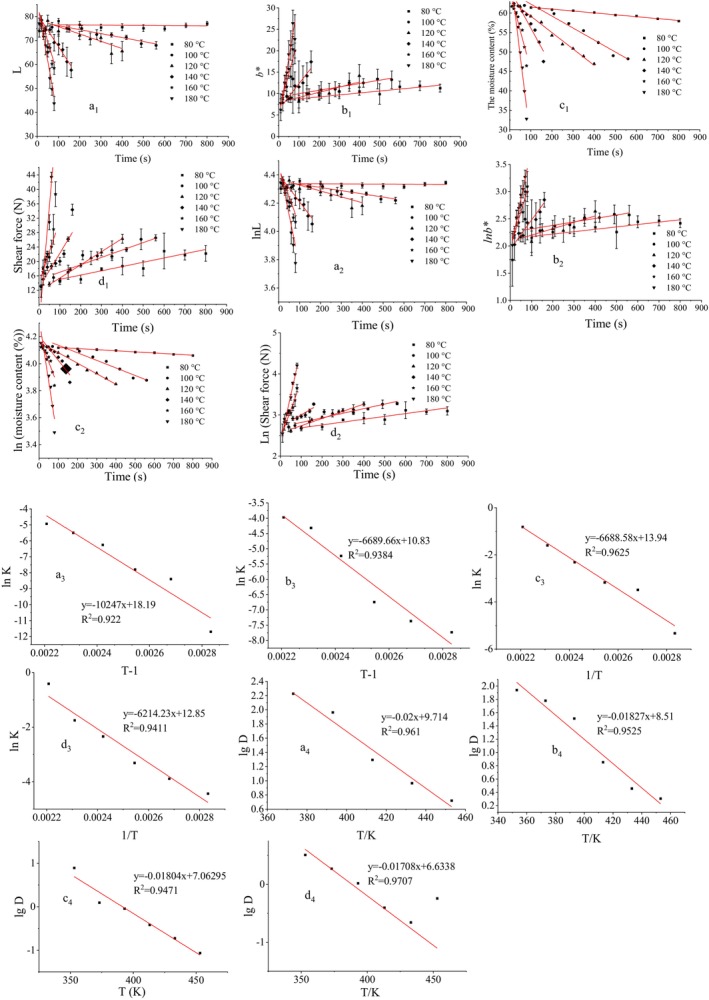
Zero‐order reaction kinetics (a1, b1, c1, d1) and first‐order reaction kinetics (a2, b2, c2, d2) of brightness L*, yellowness b*, water content, and shear force, Arrhenius diagram (a3, b3, c3, d3) and *Z* value diagram (a4, b4, c4, d4) of brightness L*, yellowness b*, moisture content, and shear force changes.

Color is an important indicator of the quality of fried foods and directly affects consumers' acceptance of products (Xu et al., 2020). During stir‐frying, the color changes with high‐temperature dehydration, Maillard reaction (Dong et al., 2023), and caramelization reaction. The brightness of the fried pork tenderloin during the thermal accumulation process is shown in Figure 2a_1_,a_2_.

The experimental results show a linear relationship between brightness L* and time at a specific temperature. With the increase in stir‐frying temperature and cooking time, a hot accumulation of pork tenderloin occurred, and the brightness L* gradually decreased with an increase in stir‐frying time. In this experiment, the initial L* value is 76, which can be reduced to 44 when heated at 180°C, indicating that the color of pork tenderloin will gradually darken during the stir‐frying process. The result is consistent with the results of (Zhang, 2018).

According to Equations (1) and (2), the reaction rate constant *k* and *R* ^2^ of the change of brightness L* of the heat accumulation of pork tenderloin at different oil temperatures are listed in Table 2.

**TABLE 2:** Thermal accumulation phenomenon during deep stir‐frying, brightness L*, yellowness value b*, shear force, and moisture content rate constant *k* and *R* ^2^.

	Temperature/°C	0‐order reaction kinetics		Average coefficient of determination	1st‐order reaction kinetics		Average coefficient of determination
		Reaction rate constant/k/s^−1^	Coefficient of determination/*R* ^2^		Reaction rate constant/k/s^−1^	Coefficient of determination/*R* ^2^	
Luminance L* rate	80	6.40E−04	0.871	0.8565	8.40E−06	0.871	0.8912
	100	1.65E−02	0.92		2.27E−04	0.961	
	120	3.05E−02	0.794		4.16E−04	0.805	
	140	0.1366	0.933		1.94E−03	0.95	
	160	0.3023	0.741		4.14E−03	0.815	
	180	0.4929	0.88		7.27E−03	0.945	
Yellow b* reaction rate	80	4.55E−03	0.889	0.8908	4.40E−04	0.874	0.8847
	100	7.88E−03	0.843		6.35E−04	0.773	
	120	1.20E−02	0.807		1.18E−03	0.845	
	140	6.02E−02	0.881		5.36E−03	0.925	
	160	0.1838	0.941		1.34E−02	0.935	
	180	0.2861	0.984		1.89E−02	0.956	
Moisture content reaction rate	80	4.88E−03	0.9893	0.9437	8.17E−05	0.9879	0.9267
	100	3.08E−02	0.97		5.60E−04	0.9615	
	120	4.22E−02	0.9876		7.81E−04	0.9919	
	140	9.98E−02	0.9		1.79E−03	0.8733	
	160	2.03E−01	0.8633		3.71E−03	0.8345	
	180	4.45E−01	0.9519		9.22E−03	0.9108	
Shear forces reaction rate	80	1.19E−02	0.8741	0.8745	6.90E−04	0.8094	0.8451
	100	2.05E−02	0.9142		1.12E−03	0.897	
	120	3.66E−02	0.972		1.75E−03	0.8329	
	140	9.56E−02	0.8178		2.98E−03	0.8213	
	160	1.74E−01	0.7873		1.40E−02	0.7861	
	180	6.68E−01	0.8816		2.30E−02	0.9238	

The determination coefficients of brightness L* change in the thermal stacking process for the zero‐order and the first‐order reaction are 0.8565 and 0.8912, respectively. At different oil temperatures, the determination coefficients *R* ^2^ of brightness L* changes in the thermal accumulation of pork tenderloin was greater than 0.7, indicating that the regression effect was significant. If *R* ^2^ is closer to 1, the higher the reference value of the relevant prediction model. Therefore, depending on the relative magnitude of the determination coefficient, the change in brightness L* of thermal accumulation is considered the first‐order reaction kinetics.

According to Equation (3), the relationship diagram was drawn between ln*k* vs. *T* ^−1^. Then, the activation energy of the process can be derived from the straight‐line slope. The higher the activation energy, the stronger the thermal sensitivity of the color change in the thermal accumulation process, and the more representative it is in describing the color change. The *Z* value of the process can be calculated by drawing the relationship between lg*D* vs. *T* according to Equation (5), and the result is shown in Figure 2a_3_,a_4_.

Figure 2c presents that the Arrhenius diagram with the change in brightness L* has an excellent linear fit (*R* ^2^ = 0.922). From this straight line, the activation energy *E*_*a*_ value of the cooking process was calculated to be 85.19 kJ/mol. The result is similar to the data of Yu et al. (2018). Figure 2a_4_ shows that the *Z*‐value graph of the change of the brightness L* also has an excellent linear fit (*R* ^2^ = 0.961). The *Z* value of the process obtained by the regression line is 50°C.

#### 3.1.2 Kinetic analysis of yellowness b* during thermal accumulation

Food has different colors because it contains various types and quantities of pigments, while the color belongs to external sensory quality. The changes can reflect consumers' most intuitive feelings about cooking products. The stir‐frying process is accompanied by high‐temperature dehydration, Maillard, and caramelization reactions. The shell will produce an attractive golden color, the quality of which determines the purchasing power of consumers. The change in yellowness color during the thermal accumulation process of fried pork tenderloin is shown in Figure 2b_1_,b_2_.

The experimental results show that the yellowness b* has a linear relationship with time at a specific temperature. When the pork tenderloin heats up as the stir‐frying temperature and cooking time increase, the yellowness b* increases gradually with the increase in stir‐frying time. The result is consistent with the findings of Mondal and Dash (2017). With the increase in stir‐frying time and the temperature of the oil bath, the luminosity (L*) value decreased, which is accompanied by an increase in the b* value.

Based on Equations (1) and (2), the change of yellowness b* at a different time and temperature, as shown in Figure 2b_1_,b_2_, was fitted and analyzed. The reaction order rate constants *k* and *R* ^2^ of the change of yellowness b* of pork tenderloin at different oil temperatures are listed in Table 2.

The average determination coefficient *R* ^2^ obtained by the fitting analysis with zero‐order and first‐order reaction models is 0.8908 and 0.8847, respectively, showing that the fitting degree of the two kinetic models is almost the same. When the reaction rate of reactants is slow, there is no significant difference between zero‐order and first‐order reactions (Li et al., 2015).

Therefore, the yellowness b* of fried pork tenderloin may be due to the slow change of rate, which leads to little difference in the fitting degree between the two reaction orders. Here, the first‐order kinetic model can be considered to explain.

By using Equations (3) and (5), the activation energy (*E*_*a*_) and *Z* value can be calculated from the yellowness b* change at a different time and temperature, as plotted ln*k* vs. *T* ^−1^ and lg*D* vs. *T* in Figure 2b_3_,b_4_. Arrhenius regression line and *Z*‐value graph show the change of yellowness b* at a different time and temperature. Regression was performed on the ln*k*–*T* ^−1^ and lg*D*–*T* changes of the yellowness b*, respectively. The Arrhenius (*R* ^2^ = 0.9384) and the *Z*‐value (*R* ^2^ = 0.9525) fit well. From the regression line, the *E*_*a*_ value was calculated as 55.62 kJ/mol, and the *Z* value was 54.73°C.

### 3.2 Kinetic analysis of water content change during the thermal accumulation

The moisture content is one of the main factors affecting the quality of fried food shells (Das et al., 2010). As an essential part of meat products, moisture content directly affects the tenderness of meat products (Wu et al., 2016). Figure 2c_1_,c_2_ presents the changes in water content through the heat accumulation process of fried pork tenderloin. The stir‐frying process is accompanied by high‐temperature dehydration. At 80 ~ 180°C, the moisture content decreases exponentially with the stir‐frying time (Figure 2c_1_), and the higher the stir‐frying temperature, the lower the water content. A partial vapor pressure difference between the product and oil causes moisture loss. These findings are consistent with data from various literature on food stir‐frying (Yu et al., 2020).

Figure 2c_1_,c_2_ presents a fitting analysis of the change in water content at different times and temperatures based on Equations (1) and (2). Table 2 lists the relevant data obtained from the analysis. The average determination coefficient *R* ^2^ obtained from a fitting study of the zero‐order reaction kinetic model is 0.9437, and the average decision coefficient *R* ^2^ obtained by fitting and analyzing the first‐order reaction kinetic model is 0.9267. Since the zero‐order kinetic model is more fitting, it is selected for interpretation.

By using Equations (3) and (5), the activation energy (*E*_*a*_) and *Z* value of water content change at a different time and temperature, which can be calculated by plotting the curve between ln*k* vs. *T* ^−1^ and lg*D* vs. *T*, as shown in Figure 2c_3_,c_4_. From the regression line, the *E*_*a*_ value of this process is 55.61 kJ/mol (*R* ^2^ = 0.9625), and the *Z* value is 55.43°C (*R* ^2^ = 0.9471). The measured correlation coefficient *R* ^2^ is greater than 0.9, showing that the established linear regression equation is effective. The *Z* value of the moisture content of fried pork tenderloin is much higher than the *Z* value of the overheat quality factor of water (30°C) (Li et al., 2017).

When the stir‐frying temperature elevated from 80 to 180°C, the kinetic constant *k* increased, indicating that the water loss of pork tenderloin increased with heating, and the temperature loss increased, as a result of the intensified denouement of myofibril associated with pork water‐retention when the temperature of heating increased. The lower water content during stir‐frying accelerates the Maillard reaction, which produces colored pigments that lead to browning. Thus, the higher the temperature, the faster the pork tenderloin burns.

### 3.3 Kinetic analysis of shearing force during thermal accumulation

Tenderness is one of the most important indicators for assessing the edible quality of meat products (Sasaki et al., 2014). Many physical and biochemical reactions occur in the meat during heating, in which many qualities change significantly. Among them, the tenderness change largely determines the consumer's purchase trend. The effect of heating temperature and time on meat tenderness is most significant. The objective assessment of meat tenderness can be measured using instruments, and the most common is the cutting force, also known as the shearing force.

The change of shear force during the thermal accumulation of fried pork tenderloin is shown in Figure 2d_1_,d_2_. Pork tenderloin tends to increase the shearing force as the stir‐frying time increases, especially when the stir‐frying temperature is between 140 and 180°C. The shearing force changes rapidly. The increase in shear force is due to the water loss of pork tenderloin, and the denaturation and aggregation of myofibrillar protein in the meat will make the meat hard, while the collagen gel dissolution and gelation will make the meat tender.

The shear force changes at different times and temperatures, as shown in Figure 2d_1_,d_2_, are fitted and analyzed using Equations (1) and (2). Table 2 presents the results of this analysis. The average determination coefficient *R* ^2^ was found to be 0.8745 by fitting analysis of the zero‐order reaction kinetic model. Using the first‐order reaction kinetic model fitting analysis, the average decision coefficient *R* ^2^ was obtained as 0.8451. The results show that the zero‐order kinetic model has a high degree of fitting, so the model is chosen to explain the dynamics of the changes in shear force.

By Equations (3) and (5), the activation energy (*E*_*a*_) and *Z* value of the shear force change at a different time, and temperature can be calculated by plotting curves between ln*k* vs. *T* ^−1^ and lg*D* vs. *T*. The result is shown in Figure 2d_3_,d_5_.

As shown in Figure 2d_3_,d_5_, ln*k* vs. *T* ^−1^, and lg*D* vs. *T* curves explain that changes in the shear force are regressed. The *E*_*a*_ value obtained during this process is 51.67 kJ/mol (*R* ^2^ = 0.9411), and the *Z* value is 58.55°C (*R* ^2^ = 0.9707). Protein denaturation is the key factor in the formation of tenderness. The shear force *E*_*a*_ value was found to be lower than the activation energy (200 ~ 600 kJ/mol) of protein denaturation (Ovissipour et al., 2013), while the *Z* value is higher than the value of protein heat denaturation (5 ~ 10°C). The results show that the factors that cause the change in the shear force value are not only the protein state but also accompanied by other factors.

### 3.4 Sensory evaluation conclusion

**FIGURE 3:**
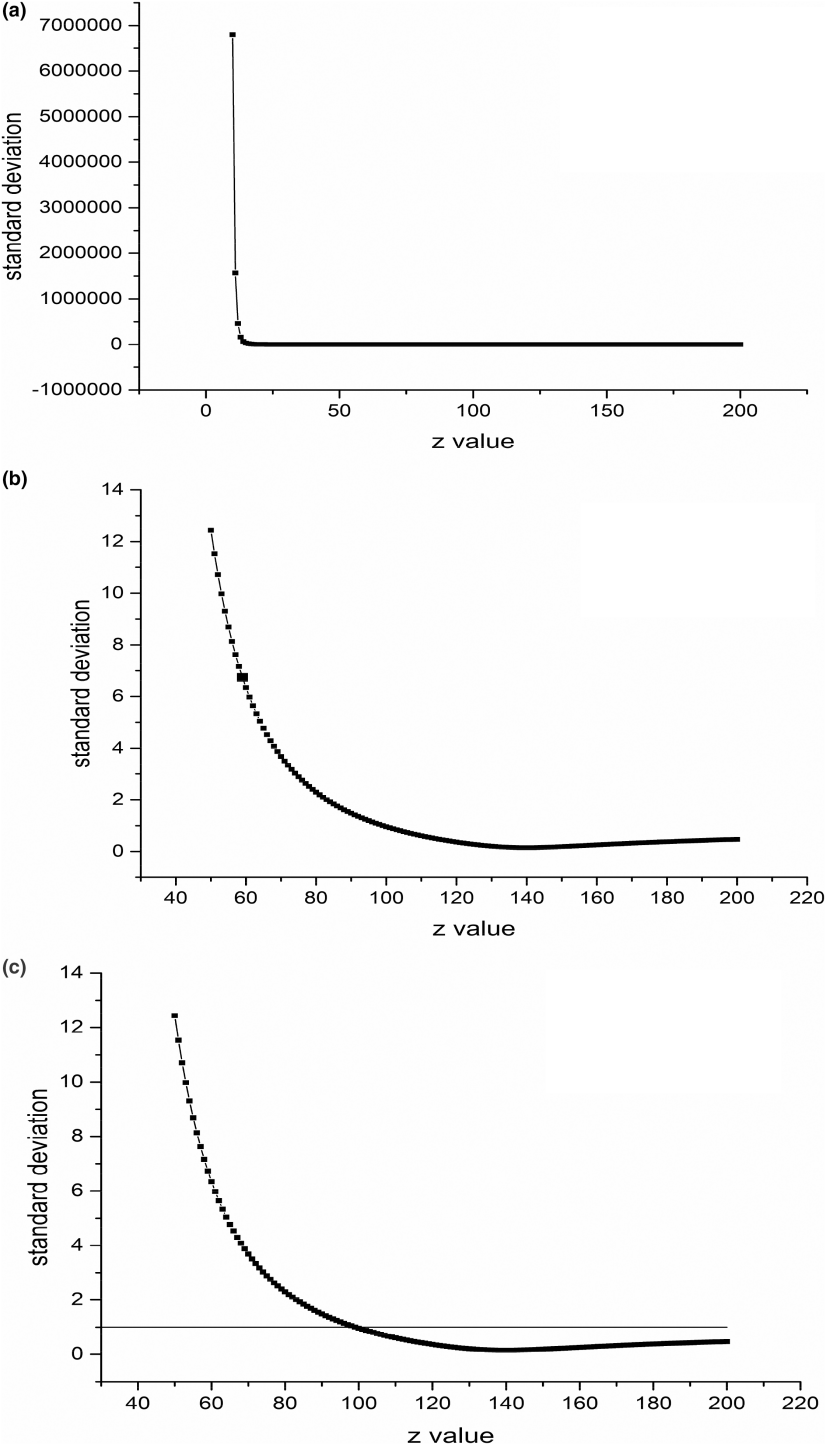
Standard deviation σ(*O*_*s*_) of *O*_*s*_ at different *Z* values (a) (b); Determination of the *Z*‐value of the heat accumulation process by sensory (c).

Table 3 presents the data on sensory tests. The sensory scores in Table 3 were used to fit the score data, in which a score of 60 was used for interpolation. The corresponding times of 60 score at four different temperatures from 120 to 180°C were 218.82, 169.51, 117.55, and 83.04 s, respectively. By using Equation (6), Matlab gives *Z* value, the reference temperature is selected to 110°C. The surface superheat value *O*_*s*_ corresponding in t_60_ at four temperatures, and the standard deviation of *O*_*s*_ at each *Z* value *σ*(*O*_*s*_) are shown in Figure 3a,b.

**TABLE 3:** Sensory scores for each group of temperatures during the stir‐frying process.

120	Time/s	30	60	90	120	150	180	210	240	270	300
	Average	58.50	64.13	70.00	81.63	84.63	80.25	71.50	60.25	49.13	42.25
	Standard deviation	12.74	13.96	14.66	9.10	5.24	9.56	9.59	9.65	12.11	9.35
140	Time/s	20	40	60	80	100	120	140	160	180	200
	Average	57.25	63.63	68.13	81.13	83.38	84.00	74.75	64.88	54.63	43.75
	Standard deviation	13.22	14.22	15.05	9.83	7.23	6.93	9.88	6.60	4.60	7.91
160	Time/s	15	30	45	60	75	90	105	120	135	150
	Average	51.13	61.25	69.38	81.50	83.75	82.75	70.25	58.00	49.13	41.38
	Standard deviation	16.63	14.33	10.16	8.50	6.94	10.58	10.00	9.91	9.80	10.66
180	Time/s	10	20	30	40	50	60	70	80	90	100
	Average	53.38	60.38	70.25	80.13	81.13	83.50	74.38	63.50	52.00	41.75
	Standard deviation	16.60	12.93	9.56	12.82	10.11	10.74	13.77	13.27	9.01	11.18

Assuming different *Z* values, use Equation (6) to calculate the surface superheat value *O*_*s*_ at each sampling time at each temperature. The reference temperature is based on literature (Purlis & Salvadori, 2007) and experimental verification. When the temperature is between 105 and 115°C, thermal accumulation begins to occur, so the reference temperature is set to 110°C. The standard deviation of the surface superheat value converted from the sensory score was calculated for each hypothesized *Z* value. The same process was followed for each temperature of a particular *Z* value. Finally, the standard deviations corresponding to the four temperatures of the *Z* value are averaged as the human sensory error *σ*(*O*_*s,z*_). The sensory error standard deviations of *Z* values from 60 to 160°C are obtained as 1.81, 0.96, 0.8, 0.7, 0.6, 0.58, 0.54, 0.51, 0.49, 0.47, and 0.45. The average *σ*(*O*_*s,z*_) value is 0.855. In Figure 3a,b, the standard deviation of *O*_*s*_ of the *Z* value is *σ*(*O*_*s*_). The minimum point is found as (140, 0.148362), and after adding an intercept of 0.855, it comes as 1.0073098. By using *σ*(*O*_*s*_) = 1.0037098 to intercept the data in Figure 2b, the intersection point *Z* value comes as 99°C. The mature quality factor *Z*_M_ of pork tenderloin is about 10°C, which is stable and unaffected by changes in the quality of meat materials (Xu, 2019). The difference between the *Z* value of this experiment is large, indicating overheating.

At the same time, there is a large difference between the *Z* value of this intersection point and the above *Z* value range (50 ~ 58.55°C), showing the difference in sensitivity between human sensory and physical and chemical measurement methods. From the reported results (Li, 2015), it is found that the measurement results of the instrument and the sensory measurement of the human being are also quite different, and further research is needed.

At the same time, when *Z* is 99°C, *O*_*s*,max_ shows as 5.86 min, explaining that after the surface experience of 5.86 min at the reference temperature of 110°C, the sensory organs of later generations will not accept this quality. This conclusion supports the existing mature value theory. The reported data (Yan et al., 2014) show that the end‐point maturity of pork tenderloin is 0.5 min. In this section, *O*_*s*,max_ is determined by a sensory test, and finally obtained the double restrictive function of mature value over the calorific value of pork loin: *Mc* ≥ 0.5 min (*Z* = 10°C, reference temperature 70°C), *O*_*s*_ ≤ 5.86 min (*Z* = 99°C, reference temperature 110°C).

In Figure 3c, *σ*(*O*_*s*_) = 1.0037098, and *Z*–*σ*(*O*_*s*_) will have two intersections. In this research, the smaller *Z* value is selected for the following reasons: (1) When the *Z* value is too large, it will cause (mature value) *M* value/(superheat value) *O* value and other dynamic parameters are too small or even less than the human sensory evaluation error, and the significance is limited; (2) From the perspective of engineering application, the *Z* value is small, and the temperature is related to the *M* value/*O* value. Through the sensory error analysis, the standard deviation of the sensory error is obtained by intercepting *Z*–*σ*(*O*_*s*_) to get a smaller *Z* value with practical significance and application value.

The physical and chemical changes formed under thermal accumulation are summarized in the following three points: (1) dry evaporation is considered to be the space where the temperature is higher than the boiling point; (2) the physical properties, hardness, and shear force are large (van Koerten et al., 2015); (3) Color changes, such as meat color changes from red to white, gray, tan, or even black (Portanguen et al., 2014). Figures S1 and S2 show the relevant stereomicroscope photograph of this analysis.

## 4 CONCLUSION

This present analysis has shown that during the thermal accumulation of pork tenderloin, the stir‐frying temperature and stir‐frying time have an essential effect on the product quality, mainly browning. During the stir‐frying process with different temperatures and times, the pork tenderloin has a more obvious change of color, resulting in a decrease in the value of L* and an increase in the value of b*. At the same time, due to the loss of water during the stir‐frying process, the water content gradually decreases while the shearing force gradually increases.

A kinetic study of the thermal accumulation process was performed. By comparing the decision coefficients *R* ^2^ using two kinetic models, the zero‐order reaction kinetic model can better describe the changes in L* and b* during thermal accumulation, while the changes in water content and shear forces align more with the first‐order. In the reaction kinetics model, the *E*_*a*_ values of the four indicators are 85.19, 55.62, 55.61, and 51.67 kJ/mol, while the *Z* values are 50, 54.73, 55.43, and 58.55°C, respectively. This research provides data support and a theoretical basis for future research.

By using the superheat value as a thermal accumulation indicator and combining it with the sensory evaluation, the *Z* value of the human sensory overheating of pork tenderloin can be determined as 99°C, and *O*_*s*,max_ (*Z* = 99°C, reference temperature of 110°C) to be 5.86 min. At the same time, there is a large difference between the *Z* value and the *Z* value of the above four indexes obtained from 50 to 58.55°C, indicating a difference between the human senses and the instrumental measurements, and the follow‐up is worth further research.

## AUTHOR CONTRIBUTIONS

**Mingzan Zhang**: Data collection and analysis, Manuscript revision. **Yun Yang**: Data collection and analysis, Manuscript revision. **Hongwen Zhang**, experiment, Writing. **Cuiqin Li**: Conceptualization, Methodology, Supervision, Manuscript revision. **Laping He**: Writing—review & editing, Funding acquisition, Supervision. **Li Deng**: Supervision, Manuscript revision. All the authors have accepted responsibility for the entire content of this submitted manuscript and approved submission.

## FUNDING INFORMATION

This work was sponsored by the Key Agricultural Project of Guizhou Province (QKHZC‐[2021]YB184, QKHZC‐[2021]YB278, QKHZC‐[2021]YB142, QKHZC‐[2019]2382, and QKHZC‐[2016]2580), National Natural Science Foundation of China (31660010 and 31870002), Qiankehe talents project ([2018]5781 and [2017]5788‐11).

## CONFLICT OF INTEREST STATEMENT

The authors declare no conflicts of interest regarding this article.

## Supporting information


Data S1.

## Data Availability

Data will be made available on request.
