# Supplementary material for: Changes in food quality and characterization under thermal accumulation conditions during Chinese cooking
Source: Food Sci Nutr. 2023 Dec 29;12(3):2081–92. doi: 10.1002/fsn3.3908 (PMC10916625; doi:10.1002/fsn3.3908)

**Supporting Information**


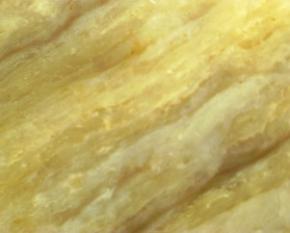

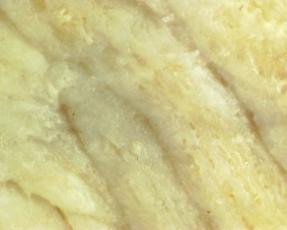

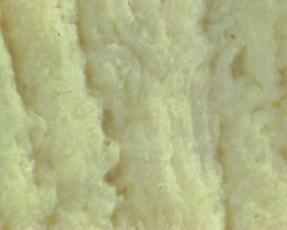

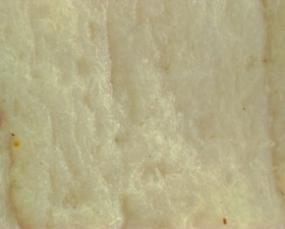

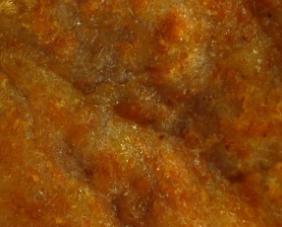

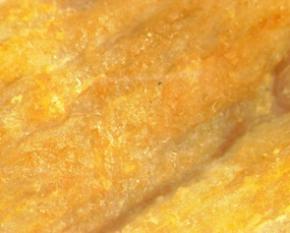

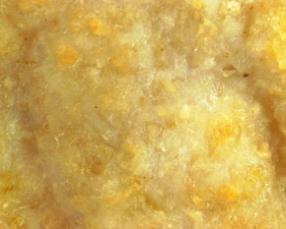

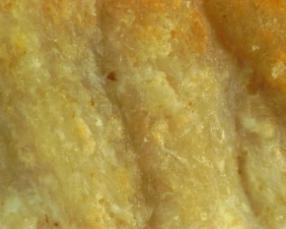


**FIGURE S1**. Stereomicroscope photo (the meat slice is 10, 20...80s in 180 degree oil)

**FIGURE S2**. Stereomicroscope photo (cross-sectional view) (meat slices fried at 180 degrees 10, 20...80s


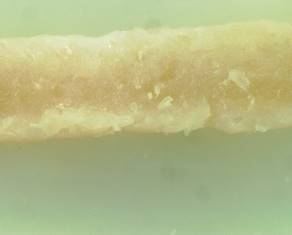

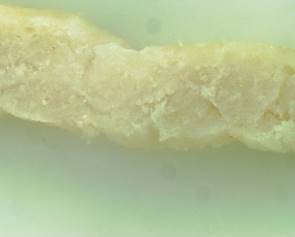

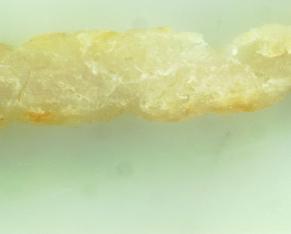

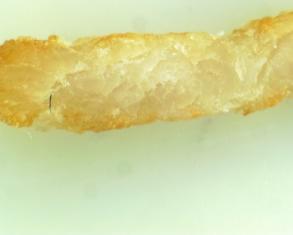

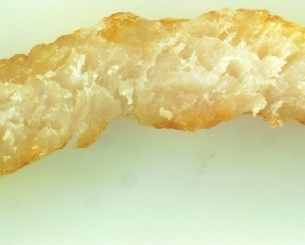

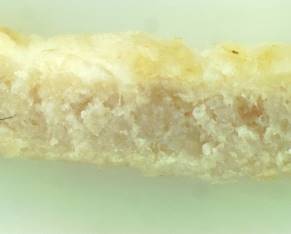

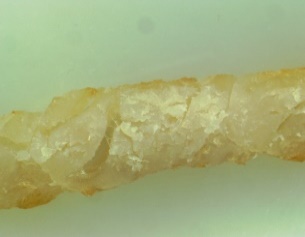

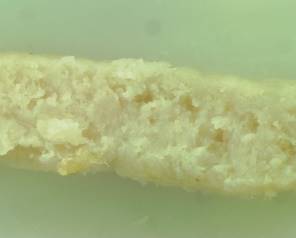

Supplement: Supplementary file 1 — Data S1. [file FSN3-12-2081-s001.docx]
